# Survival and time to initiation of adjuvant chemotherapy among breast cancer patients: a systematic review and meta-analysis

**DOI:** 10.18632/oncotarget.23086

**Published:** 2017-12-07

**Authors:** Qiao-Hui Zhan, Jian-Qin Fu, Fang-Meng Fu, Jie Zhang, Chuan Wang

**Affiliations:** ^1^ Department of General Surgery, Fujian Medical University Union Hospital, Fujian Province, Fuzhou, China

**Keywords:** time, adjuvant chemotherapy, breast cancer, suvival, meta-analysis

## Abstract

The relationship between survival and time to the start of adjuvant chemotherapy (AC) among breast cancer patients is unclear. In order to illustrate the effect of delaying the initiation of AC on survival we have undertaken a systematic review and meta-analysis.

We identified 12 available studies in the meta-analysis including 15 independent analytical groups. This meta-analysis showed that a 4-week delay before AC was associated with a significantly worse overall survival (OS)(HR=1.13; 95% confidence interval [CI], 1.08–1.19) and disease free survival (DFS)(HR=1.14; 95%CI, 1.05–1.24). Two studies categorized patients into hormone receptor-positive, ERBB2-positive, and triple-negative breast cancer (TNBC) patients according to the clinicopathological features of breast cancer. The HRs for OS between waiting time (WT) ≤30 days and 31–60 days in the subgroups were extracted and analyzed. The analysis demonstrated that a WT of 31–60 days was related to worse OS among patients with TNBC (HR, 1.26; 95% CI, 1.08–1.48), but had no significant effect on OS among those with hormone receptor-positive (HR, 1.02; 95% CI, 0.89–1.15) or ERBB2-postive (HR, 0.95; 95%CI, 0.79–1.14) tumors.

In this meta-analysis of the eligible literatures reviewing the time to AC, a longer waiting time to adjuvant chemotherapy may lead to worse survival in breast cancer patients, especially in TNBC patients.

## INTRODUCTION

In recent decades, the incidence of breast cancer has gradually increased and breast cancer has become the top killer of women [1]. Many randomized trials have demonstrated that breast cancer patients obtaine survival benefits from adjuvant chemotherapy [2]. Compared to patients who do not receive chemotherapy, adjuvant chemotherapy decreases by 30% to 40% the risk of breast cancer mortality [3]. Adjuvant chemotherapy is commonly used to improve survival and to reduce the risk of recurrence in breast cancer patients, particularly in patients with primary tumors that are large, estrogen receptor (ER) negative, high grade, and with lymph nodes involvement [4]. Most breast cancer patients start adjuvant chemotherapy within a few weeks after surgery, but it is still unclear whether a delay in the initiation of chemotherapy will lead to adverse outcomes. There are reasons to believe that early initiation of chemotherapy after surgery might improve survival, it has been known since the late 1970s that surgical trauma and tumor removal might result in an increased number of circulating tumor cells and an accelerated growth of micrometastases [5]. Clinically, the majority of breast cancer patients will eventually receive AC, however, the optimal time to initiate AC is undefined. Some clinicians have suggested that a 3-month delay in time to start AC seems to be associated with a substantial decrease in the efficacy of systemic therapy [6]. Some studies have researched the effect of delays in initiating AC after breast cancer surgery. Four of these studies reported no association between initiation of AC and survival [7–10]. However, nine other studies found worse survival (disease free and/or overall) in patients starting AC more than four weeks [11], 35 days [12, 13], 60 days [14], 10 weeks [15], 12–24 weeks [16], three months [17–19], and five months [20] after surgery. In order to explore the impact of delaying the initiation of AC on the survival of breast cancer patients, we have performed a systematic review of all the relevant studies and undertaken a meta-analysis of the available literature.

## METHODS

### Searches

**Figure 1:**
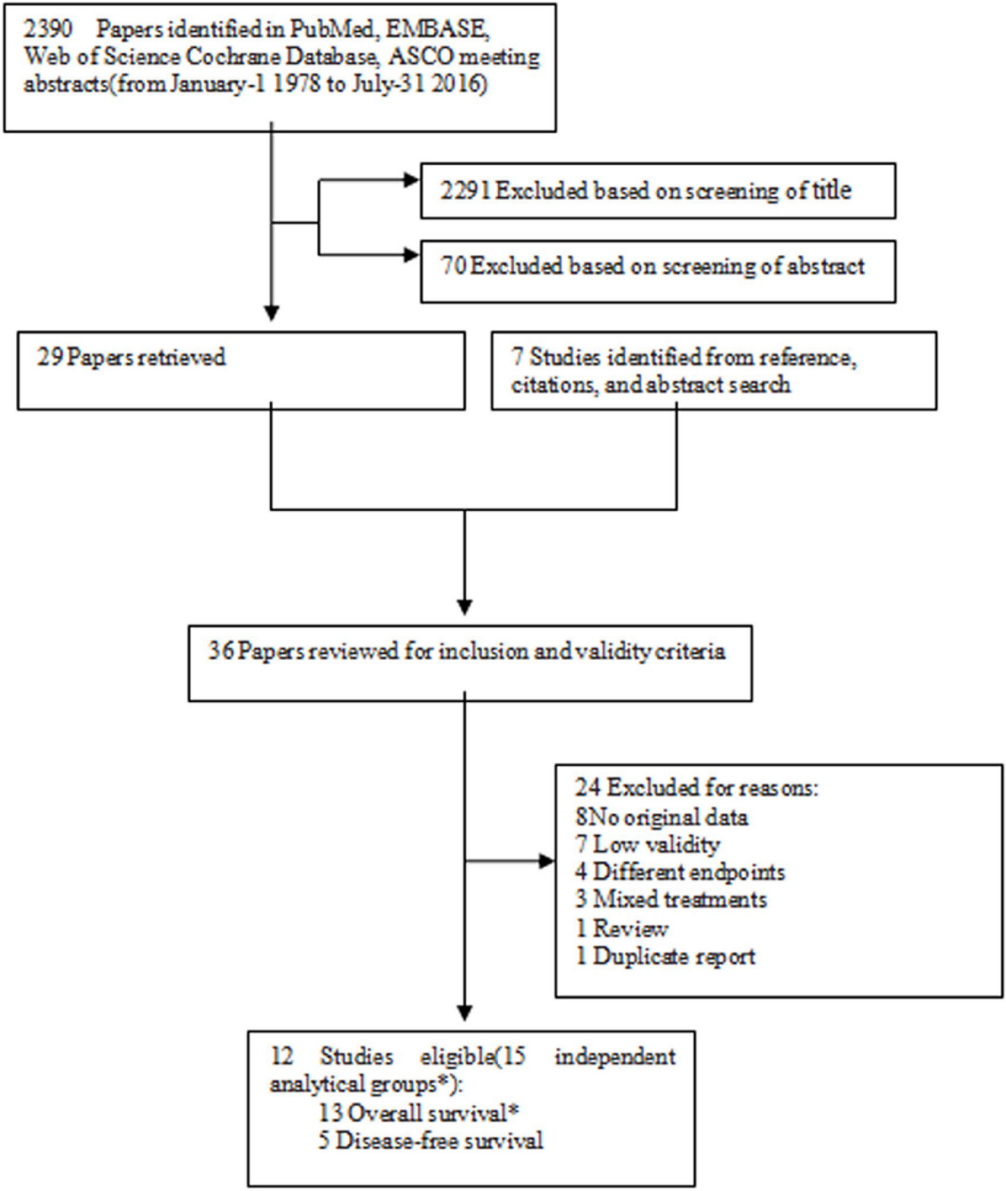
Flowchart of the study selection strategy *Two studies include more than one analytical groups of overall survival.

Figure 1 presents the detailed procedures of the literature search and screening. All of the potentially relevant literature in PubMed, Google Scholar, EMBASE, Cochrane Database, and Web-of-Science from January-11978 to July-31, 2016 was searched using the key words; timing or time and adjuvant and chemotherapy or chemotherapeutic and breast cancer and survival. To yield more relevant articles, abstracts available from the online proceeding were searched for newly completed articles, especially those of annual meetings of the American Society of Clinical Oncology (ASCO) from 2007 to 2016. Furthermore, we reviewed the reference lists from relevant studies to identify studies not identified in the original search. The basic procedure of the meta-analysis was performed as previously described, and the procedure adheres to the standards of quality for reporting meta-analysis [21–23]. In order to reduce the effect of any publication bias, full-text articles and meeting abstracts, were eligible for inclusion.

### Selection

All eligible literatures were required to satisfy the following criteria:

1. All of the breast cancer patients were treated with AC, and the time interval after surgery and before beginning AC was documented.

2. The relationship between the time between surgery and starting AC and the subsequent outcome of breast cancer patients was reported.

3. Disease-free survival (DFS), relapse-free survival (RFS), event-free survival (EFS), or overall survival (OS) was used as the outcome for breast cancer patients. The hazard ratio (HR) of DFS, EFS, RFS, or OS with 95% confidence intervals (CIs) when they were reported or when there was sufficient data to calculate these values.

4. To reduce the impact of confounding factors between comparison groups, articles included in the final study cohort were identified to satisfy the following criteria: (1) the relevant prognostic factors were sufficiently described between compared groups; (2) either the compared groups or the reported results were balanced for the relevant prognostic factors [21].

### Exclusion criteria

1. Studies were excluded by using nonstandard forms of AC. For instance: neoadjuvant chemotherapy, dose-dense chemotherapy or perioperative chemotherapy.

2. Studies that examined the impact of additional adjuvant therapies (e.g, radiotherapy or endocrine therapy) were excluded.

### Data extraction and conversion

According to the procedure described previously [21, 23, 24], we performed this step with several modifications. An HR for two survival measures (OS and/or DFS) was used to measure the effect in all studies. Since the definition of EFS and RFS was similar to DFS, EFS and RFS were treated as DFS for most studies. The representations of Waiting Time (WT) were categorized disparately in the available studies. In order to provide a common representation for evaluation of the results of individual studies, the WT effect was converted to a regression coefficient (β) and its standard error (SE) corresponding to a continuous representation per 4-weeks of WT. In our study, 4-weeks of WT might be the most suitable cut-off time. Since there is a lack of original data to calculate the sensitivity and specificity, we could not use a ROC curve to evaluate the best cut-off. Most eligible studies used 4-weeks as cut-off. Four of nine studies were based on a cut-off of 4-weeks for OS, as were 3/5 studies for DFS in this meta-analysis. Moreover, a one week delay would lead to a very small impact on survival which could not be detected. And when the cut-off time was extended to 4-weeks, an obvious effect on survival of delayed AC could be found. Therefore, we chose 4-weeks as the cut-off for the meta-analysis. Each WT category assigned a central value in each study. For studies with 2 WT groups, the two groups were defined as “before n weeks” and “after n weeks”, thus we treated the reference time level as n/2weeks and the exposure time level as n/2+n weeks. The weekly β was calculated as ln(HR)/(Xn-X0), and the corresponding SE of β was calculated as (ln[upper of 95% CI]-ln[lower of 95% CI])/([Xn-X0] × 3.92); where CI is the confidence interval, Xn denotes the exposure at group n level, and X0 denotes the exposure of the reference group. All the time units (days, weeks, or months) were transformed to “weeks” and “n” in the Xn denotes the number of weeks. If only a *P*-value was provided, the SE was calculated with the “test-based” method; SE = (ln[HR])/Zp, where *Zp* is the value of a unit-normal test (e.g., *Zp* = 1.96 when *P* = 0.05, 2-tailed test). For the studies with more than two WT groups, weighted least-squares linear regression of the ln(HR) at every exposure level in a study was used to estimate the summary β with weights equal to the inverse of the variance of the HR estimates [25, 26]. The dependent variable for the regression was log(HR) corresponding to each study. The summary measures of HR per 4-weeks of delay from each study can be interpreted as the incidence rate ratio for death or recurrence with each 4-weeks of additional waiting for AC, thus the summary measures presented here could be equal to eβ x 4. These estimates are based on the assumption of a log linear relationship across WTs, and are only related to the range of WTs covered in the eligible studies.

### Study quality assessment

Since all the articles included in our meta-analysis were nonrandomized, we use the 9-star Newcastle-Ottawa Scale to assess the quality of eligible studies: The 9-star Newcastle-Ottawa Scale is used for assessing the quality of nonrandomized studies in meta-analysis.

### Meta-analysis

The adjusted regression coefficients from individual studies were combined using a random-effects or fixed-effects model according to whether inter-study heterogeneity exists. We used the Q statistic and I^2^ to assess the between-studies heterogeneity [27]. A *P*-value <0.05 or I^2^ > 25% meant heterogeneity [28]. The inverse variance was used to weight individual studies. The sensitivity analysis was performed to find the potential outliers by sequentially omitting the largest studies and calculating a combined result from the remaining studies. The potential publication bias was detected in a funnel plot of log(HR) against its SE, and the degree of asymmetry was examined using Egger's test [29] (*P* < 0.05 considered to be statistically significant). We performed all of the statistical analysis by using Stata 12.0 (Stata Corporation, College Station, TX) and SPSS20.0 (SPSS Inc, Chicago, IL). A two-tailed *P* < 0.05 was considered statistically significant.

## RESULTS

### Characteristics of selected studies

The flow chart of the study selection strategy is shown in Figure 1. We selected 2,390 items published between 1989 and 2016 using the search strategy, and after reviewing their abstracts, 36 papers were potentially available. We further eliminated 24 reports which lacked data or did not meet the high validity criteria, and were left to consider 12 eligible papers including 78,462 breast cancer patients in our meta-analysis. One study was conducted prospectively [13], 3 studies [8, 12, 30] (with 5 analytical groups), were secondary analyses of randomized controlled trials , and the remaining 8 were retrospective investigations [14–19, 31] (9 analytical groups) using hospital-or population-based data (Table 1). Because two eligible publications included more than one analytical OS group, there were 13 independent analytical groups for OS and five for DFS in this meta-analysis (Table 2).

**Table 1:** Characteristics of eligible studies

Source	Place, Data type and name	Median age, year	Meno- pausal status	Stage	Hormone receptor-positive (%)	Chemotherapy	Median FU	Sample size	Study qualiy^*^	Adjustment for covariates
Pronzato *et al.* [13] 1989	Italy (Pros.)	51 yr (range, 27–70)	Mixed	Operable(LN+)	NR	CMF	37 months	229	7	Age, nodes status, menopausal status, cycle number, individual dose, intensity
Colleoni *et al*.[30] 2000	Multicenter(CT, IBCSG)	78% pts ≥40 yr	Pre.	Operable(LN+)	87.4	CMF	7.7 years	1,788	8	Age, size, nodalstatus, vessel invasion, and institution
Kerbrat *et al*.[31] 2005^**^	France(Retros., FASG)	NR	NR	Operable	NR	Anthr.-based	9 years	2,602	7	Multivariate, adjustmen;adjusted factors not reported
Cold *et al*. [8] 2005 (I)	Denmark(CT, DBCG)	53% pts <46 yr 43% pts 46–55 yr 3% pts >55 yr	Mixed	Operable	77	Classical CMF	NR	352	6	Age, tumour size, nodes status, histological type, grade, hormone receptor status, andadjuvant irradiation
Cold *et al*. [8] 2005 (II)	Denmark(CT, DBCG)	40% pts <46 yr40% pts 46-55 yr20% pts >55 yr	Mixed	Operable	58.3	CMF i.v.	NR	6,065	8	Age, tumour size, nodes status, histological type, grade, hormonereceptor status, andadjuvant irradiation
Cold *et al*. [8] 2005 (III)	Denmark(CT, DBCG)	40% pts <46 yr40%pts 46–55yr20% pts >55 yr	Mixed	Operable	61.8	CEF	NR	1,084	7	Age, tumour size, nodes status, histological type, grade, hormonereceptor status, andadjuvant irradiation
Hershman *et al*. [18] 2006	USA(Retros., SEER)	100%pts ≥65 yr	Post.	I–II	58	Polychemotherapy	NR	5,003	8	Age, race, live location, stage, hormonereceptor, grade, comorbid conditions, SES score, marital status, teaching hospital, surgery, and radiation
Lohrisch *et al*.[16] 2006	USA (Retros.,)	47 yr	Mixed	I–II	55.9	CMF and Anthr.-based	6.2years	2,594	8	Age, size, nodalstatus, lymphatic orvascular invasion,and anthracycline
Nurgalieva *et al*. [19] 2013	USA (Retros., BCCA)	100%Pts≥65yr	Post.	I–III	NR	Polychemotherapy	NR	14,380	8	Age, marriage status,tumor stage, size,grade, hormonereceptor status, comorbidity, year of diagnosis, SEERregion, primary surgery radiotherapy, chemotherapy, arace/ethnicity
Downing *et al*.[15] 2014(I)	UK (Retros.)	27% pts <45 yr 73% pts ≥45 yr	Mixed	I–III	NR	Polychemotherapy	NR	6,100	8	age, stage, RTreceive after CT, and year of treatment comorbidity, surgery, reconstruction
Downing *et al*.[15] 2014(II)	UK (Retros.)	25.8% pts <45 yr 74.2% pts ≥45 yr	Mixed	I–III	NR	Polychemotherapy	NR	4,266	8	age, stage, RTreceive after CT, and year of treatment comorbidity, surgery, reconstruction
Gagliato *et al*.[14] 2014	USA (Retros.,)	50 yr(range, 19– 85)	Mixed	I–III	65.4	Polychemotherapy	59.3 months	6,827	8	age, race/ethnicity, tumor size nodal status grade, LVI, type of surgery comorbidity
Farolfi *et al*.[12] 2015	Italy (CT, NCT01031030)	52 yr(range, 26–70)	Mixed	Operable	73.9	CMF-E, E-CMF and CMF	105 months	921	8	Nodal involvemt, oestrogen recept HER2 status; Ki67 value Type of adjuvant chemotherapy, menopausal status and tumour size
Chavez *et al*.[17] 2015	USA (Retros.)	53 yr	Mixed	I–III	NR	Polychemotherapy	62.7 months	24,843	8	age, sex, race/ethnicity, SES, year of diagnosis, stage, subtype, marital status, type of surgery, primary payer reconstructive surgery, whether treated at a NCI–designated cancer center
Ke-Da *et al*.[11] 2016	China (Retros.)	50 yr	Mixed	I–IIIa	71.0	Anthr.-based or Anthr.-/taxane-based.	72 months	1408	8	age, tumor size, nodal status, surgical modality, and endocrine therapy
Farolfi *et al*.[12] 2015	Italy (CT,NCT01031030)	52 yr(range, 26–70)	Mixed	Operable	73.9	CMF-E, E-CMF and CMF	105 months	921	8	Nodal involvemt, oestrogen recept HER2 status; Ki67 value Type of adjuvant chemotherapy, menopausal status and tumour size
Chavez *et al*.[17] 2015	USA (Retros.)	53 yr	Mixed	I–III	NR	Polychemotherapy	62.7 months	24,843	8	age, sex, race/ethnicity, SES, year of diagnosis, stage, subtype, marital status, type of surgery, primary payer reconstructive surgery, whether treated at a NCI–designated cancer center
Ke-Da *et al*.[11] 2016	China (Retros.)	50 yr	Mixed	I–IIIa	71.0	Anthr.-based or Anthr.-/taxane-based.	72 months	1408	8	age, tumor size, nodal status, surgical modality, and endocrine therapy

Abbreviations: Anthr, Anthracycline; BCCA, British Columbia Cancer Agency; CMF, Cyclophosphamide, methotrexate and fluorouracil; CT, Clinical trial; DBCG, Dansh Breast Cancer Cooperative Group; FASG, French Adjuvant Study Group; FU, Follow up; IBCSG, International Breast Cancer Study Group; LN+, Lymphnodes positive; NR, Not reported; Post, Postmenopausal; Pros, Prospectivestudy; Retro, Retrospectivestudy; SEER, The Surveillance, Epidemiology, and End-Resultsdatabase.^*^Evaluated by the9-star Newcastle-Ottawa Scale.

^**^The publish type of this study is a meeting abstract.

**Table 2:** Study-specific waiting time categories and outcomes

Source	WT categories	Sample size	HR (95%CI)	
			OS	DFS/RFS/EFS
Pronzato *et al*. [13] 1989	≤35 days	116	Reference	−
	>35 days	113	2.61 (1.26−5.39)	
Colleoni *et al*. [30] 2000	<21 days	599	−	0.88 (0.76−1.03)
	≥21 days	1,189		Reference
Kerbrat, *et al*. [31] 2005	< 28 days	1,614	−	0.85 (0.65−1.05)
	28–42 days	883		Reference
	>42 days	105		
Cold *et al*. [8] 2005 (I)	1–3 wks	58	Reference	−
	3–4 wks	92	0.929 (0.441−1.957)	
	4–5 wks	75	1.549 (0.761−3.149)	
	5–13 wks	127	1.588 (0.856−2.948)	
Cold *et al*. [8] 2005 (II)	1–3 wks	1,509	Reference	−
	3–4 wks	1,581	1.021 (0.903−1.155)	
	4–5 wks	1,423	0.890 (0.782−1.012)	
	5–13 wks	1,552	1.002 (0.884−1.136)	
Cold *et al*. [8] 2005 (III)	1–3 wks	188	Reference	−
	3–4 wks	305	1.218 (0.800−1.854)	
	4–5 wks	263	1.045 (0.716−1.525)	
	5–13 wks	328	1.238 (0.861−1.782)	
Hershman *et al*. [18] 2006	<1 month	2,361	Reference	−
	1–2 months	1,846	1.00 (0.88−1.14)	
	2–3 months	323	1.08 (0.85−1.36)	
	>3 months	477	1.46 (1.21−1.75)	
Lohrisch *et al*. [16] 2006	≤4 wks	993	Reference	−
	4–8 wks	1,272		
	8–12 wks	217		
	12–24 wks	112	1.6 (1.2−2.3)	
Nurgalieva *et al*. [19] 2013	≤3 months	12,748	Reference	−
	>3 months	1,632	1.53 (1.32– 1.80)	
Downing *et al*. [15] 2014 (I)	≤3 wks	557	Reference	−
	3–6wks	3,253	0.90 (0.73−1.12)	
	6–10wks	1,897	0.88 (0.70−1.10)	
	>10wks	393	1.49 (1.13−1.95)	
Downing *et al*. [15] 2014 (II)	≤3 wks	1,186	Reference	−
	3–6wks	2,279	1.00 (0.85−1.18)	
	6–10wks	652	1.10 (0.88−1.37)	
	>10wks	149	1.16 (0.80−1.67)	
Gagliato *et al*. [14] 2014	≤30 days	2,716	Reference	Reference
	31–60 days	2,994	1.05 (0.94– 1.18)	1.04 (0.94−1.14)
	≥61 days	1,117	1.19 (1.02– 1.38)	1.10 (0.97−1.25)
Farolfi *et al*. [12] 2015	≤7 wks	818	Reference	Reference
	>7 wks	103	1.14 (0.96–1.34)	1.15 (1.02–1.30)
chavez *et al*. [17] 2015	≤30 days	5,224	Reference	−
	31–60 days	12,432	0.98 (0.87–1.09)	
	61–90 days	4,765	1.01 (0.88–1.16)	
	≥91 days	2,422	1.34 (1.15–1.57)	
Ke-Da *et al*. [11] 2016	0–4 wks	871	Reference	Reference
	4–8 wks	446	1.43 (0.94−2.19 )	1.14 (0.83–1.56)
	>8 wks	91	2.02 (1.10– 3.71)	1.86 (1.19–2.90)

Abbreviations: WT, Waiting time; HR, Hazard ratio; CI, Confidence interval; OS, Overall survival; DFS, Disease-free survival; RFS, Relapse-free survival.

### Primary outcome: OS and DFS

**Figure 2:**
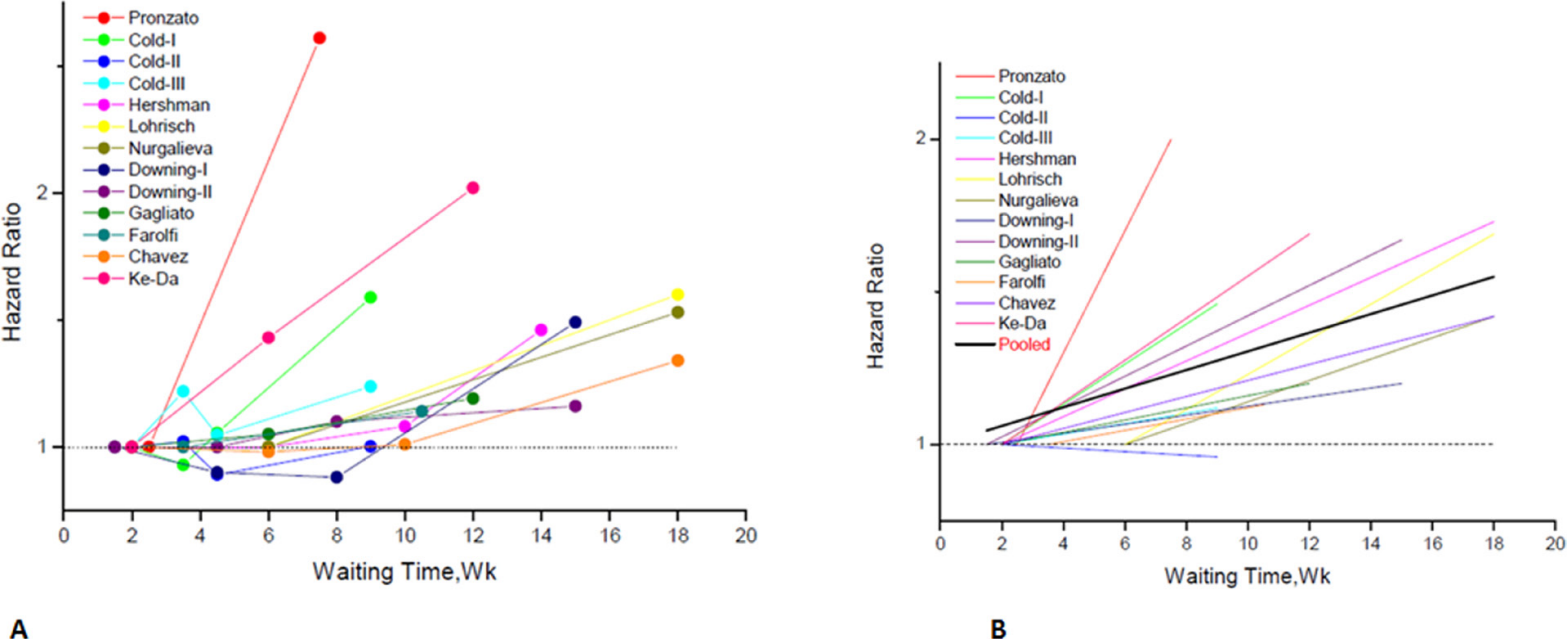
Individual hazard ratio for overall survival according to waiting time categories (**A**) The relationship between waiting time categories and overall survival in the 12 independent analytical groups. The hazard ratio (HR) represents a comparison with the first waiting time category in each study (as reference). The first author of each study is shown. (**B**) Conversion of HR estimates from the original studies to an HR per week of delay. The slope of each line represents the change in the log HR per week of delay. The line for each individual study is located over the range of waiting times. The thick line indicates the weighted average of the HRs from the individual studies. The vertical axis is on a log scale.

**Figure 3:**
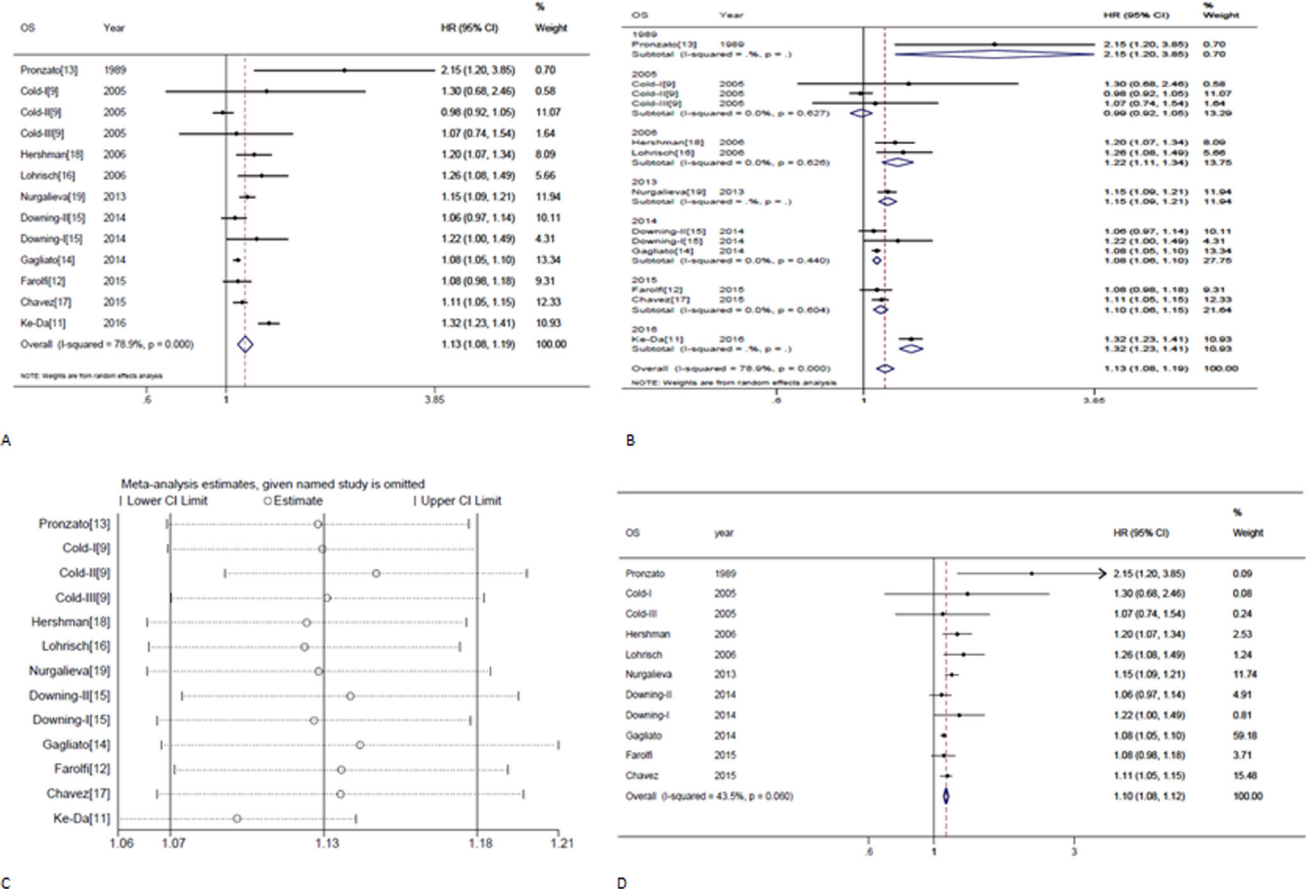
Individual study and overall hazard ratios of relationships between every 4-week delay in initiation of adjuvant chemotherapy and overall survival (**A**) Shows individual study and overall hazard ratios (HR) per 4-weeks of delay with 95% confidence interval (CI) for OS. The size of each square is proportional to the weight of the study. For the combined result, the length of the diamond represents the 95% CI of the summary. (**B**) Shows subgroup analysis for OS according to the year that the studies were published. (**C**) Shows the influence of individual studies on the pooled HR for OS. The vertical axis indicates the overall HR and the two vertical axes indicate its 95% CI. Every hollow round shape indicates the pooled OR when the left study is omitted in this meta-analysis. The two ends of every broken line represent the respective 95% CI. (**D**) Shows remaining studies after excluding Cold-II and Ke-Da studies; hazard ratios (HR) per 4-week of delay with 95% confidence interval (CI) for OS.

**Figure 4:**
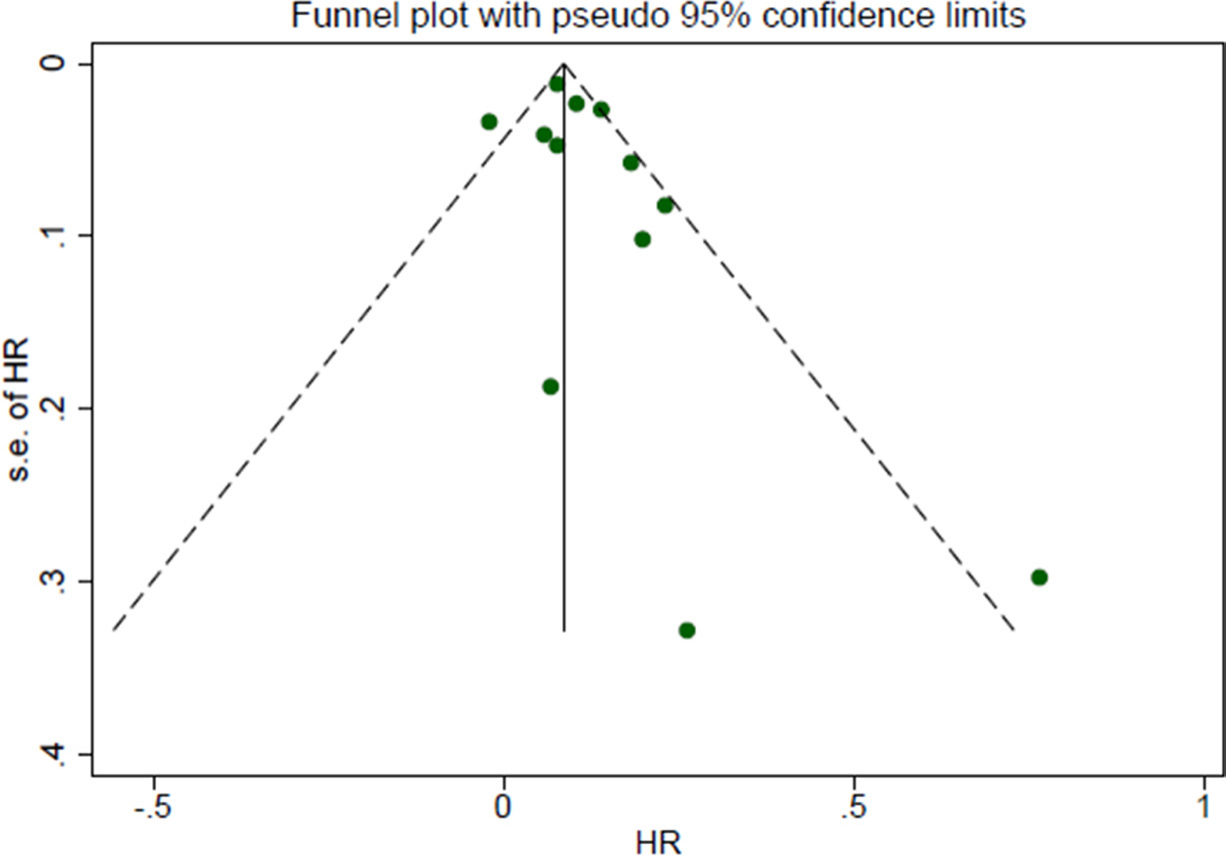
Funnel plot of the relationship between the hazard ratio and standard error of the log HR for overall survival Hazard ratio (HR) estimates are the effect per 4 weeks of waiting time. The dotted line indicates the combined HR for all studies of overall survival. Filled circles represent the 12 studies to account for potential publication bias.

The study-specific waiting time categories and the HR results in the 13 analytical groups for OS are plotted in Figure 2A. The WTs were covered by the analytical groups, and arranged from 2 to12 weeks. This figure indicates that the trend of the variation of HRs at different WTs in each study were similar, therefore, we can suppose the conversion of HRs from categories to an HR for a continuous representation by WT. Figure 2B shows every single HR corresponding to the relative decrease in survival with each 4-week increase in WT for each study. According to the different WT categories in every study, we used different methods to convert the HR estimates from the original studies to an HR per week of delay. For studies with two WT groups, the line was the same as that presented in Figure 2A. For studies using more than two categories, the HR was calculated by using meta-regression. The final HR used in our meta-analysis (HR per 4-weeks of delay) was represented by the 4-fold change of the slope of each line (by log converted HR) in Figure 2B. Figure 3A is the forest plot of this meta-analysis for the OS and shows HRs per 4-weeks of delay with 95%CIs for 13 analytical groups. The combined HR was 1.13 (95%CI, 1.08–1.19) calculated by a random-effects model. There was a significant inter-study heterogeneity for OS (*P* = 0.00; *I*^2^ = 78.9%). In order to search the resource of heterogeneity, we undertook subgroup analysis according to the year that the studies were published (Figure 3B) and the between-study heterogeneity disappeared (I^2^ = 0.0%). It demonstrated that the year was a major source of heterogeneity. Furthermore, we performed influence analysis (Figure 3C), which omits one study at a time and calculates the recombined HRs for the remainders, and the result showed no single study significantly influenced the pooled HR. However, we found that the HRs of two studies (the Cold-II study by Cold *et al*. [8] and the study by Ke-Da *et al*. [11]) obviously deviated from the combined HR. After excluding these two studies (Figure 3D) the between-study heterogeneity was significantly decreased (*P* = 0.06; I^2^ = 43.5%). It showed that the Cold-II and the study by Ke-Da were the other sources of heterogeneity. The funnel plot (Figure 4) and the Egger's test were also used to determine publication bias. There was no evidence of publication bias (*P* > 0.05) in this meta-analysis.

**Figure 5:**
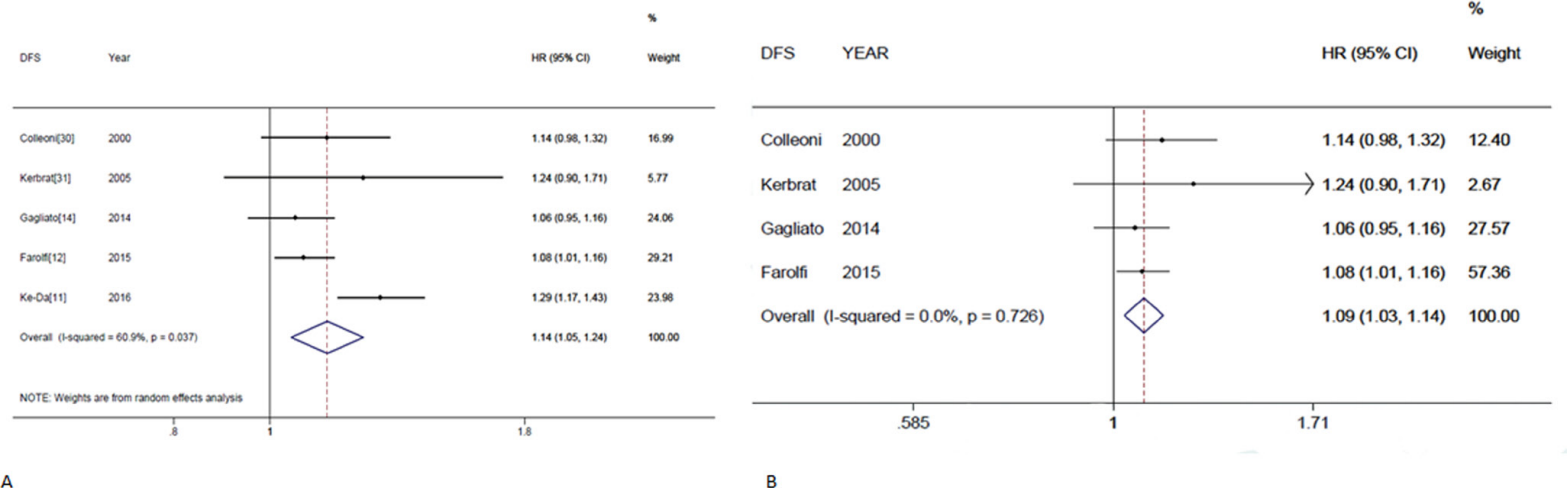
Individual study and overall hazard ratios of relationships between every 4-week delay in initiation of adjuvant chemotherapy and disease-free survival (**A**) Individual and overall hazard ratios (HR) per 4-weeks of delay with 95% confidence interval (CI) for DFS are shown. The size of each square is proportional to the weight of the study. For the combined result, the length of the diamond represents the 95% CI of the summary. (**B**) Shows remaining studies after excluding the Ke-Da study; hazard ratios (HR) per 4-week of delay with 95% confidence interval (CI) for DFS.

The same procedure of analysis was repeated for DFS, and the final forest plot for DFS is shown in Figure 5A. The combined HR was 1.09 (95%CI, 1.03–1.14) calculated by a random-effects model. There was a significant heterogeneity between the included studies (*P* = 0.037, I^2^ = 60.9%). We also performed influence analysis for DFS. After omitting the Ke-Da *et al*. study [11] the inter-study heterogeneity disappeared (Figure 5B). This indicated that the study by Ke-Da was the source of the heterogeneity

### Secondary outcome: the HRs for OS in different breast cancer subtypes

**Figure 6:**
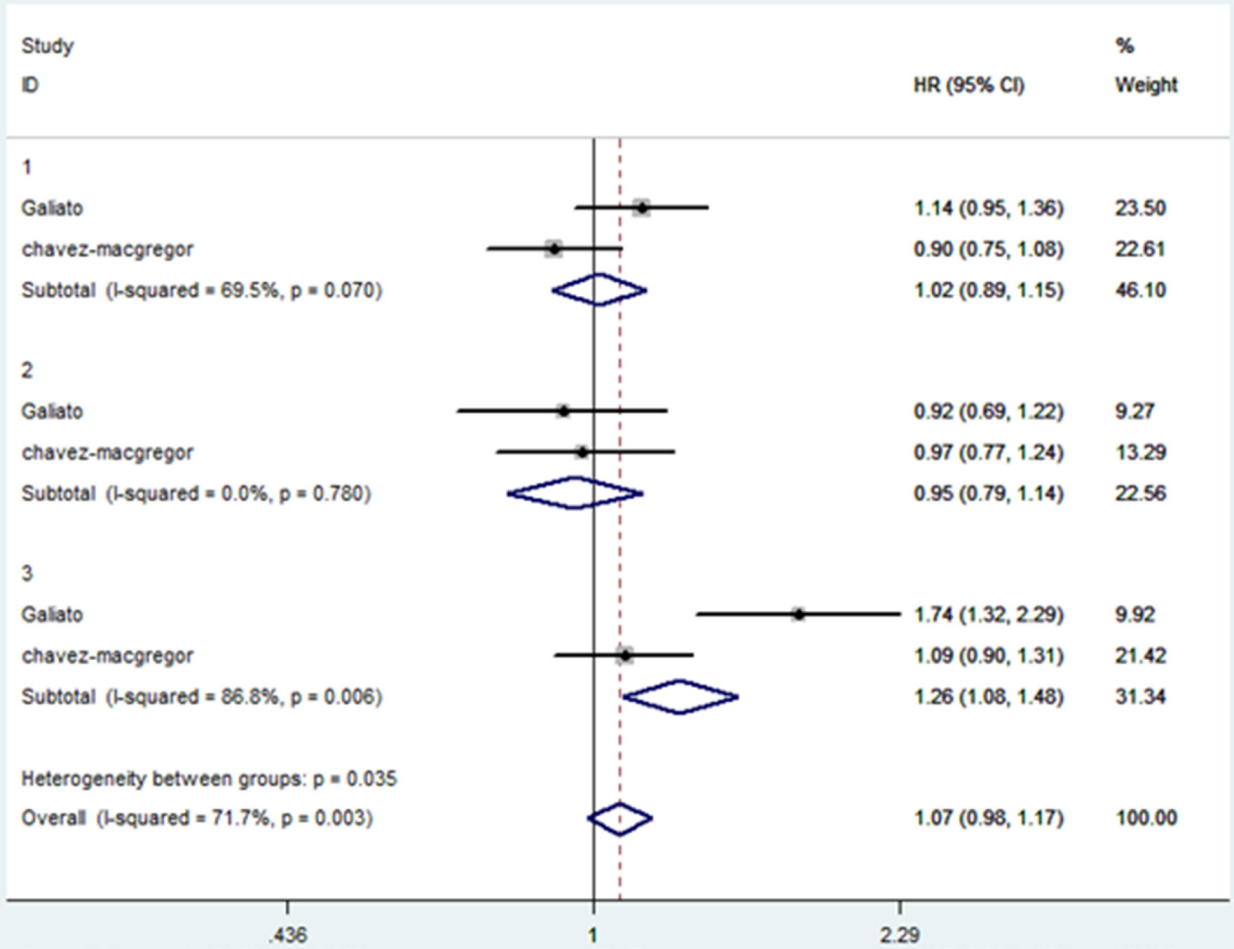
Comparison of overall survival between WT ≤30 days and 31–60 days in the three subgroups The size of each square is proportional to the weight of the study. For the combined result, the length of the diamond represents the 95% CI of the summary. Numbers indicate different subgroups.1-hormone receptor-positive breast cancer, 2-ERBB2-positive breast cancer, 3-TNBC.

**Figure 7:**
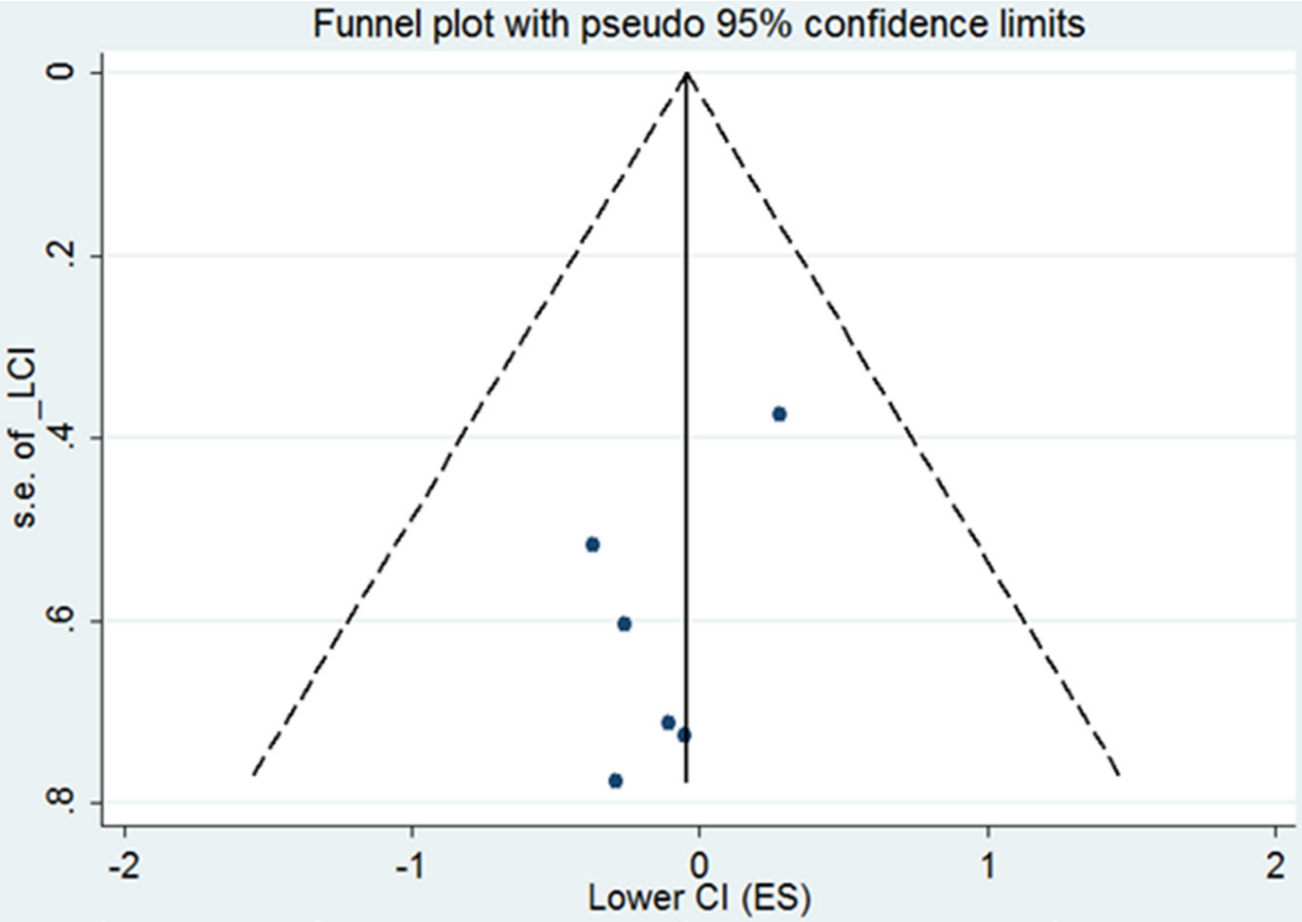
Funnel plot of the relationship between the hazard ratio and standard error of the log HR for overall survival in three different subtypes The dotted line indicates the combined HR for all studies of overall survival. Filled circles represent the 6 subgroups to account for potential publication bias.

Gagliato *et al*. [14] and Chavez-MacGregor *et al*. [17] categorized patients into hormone receptor-positive, ERBB2-positive and TNBC subgroups. The HRs for OS between WT ≤30 days and 31–60 days in the three subgroups was extracted and analyzed. The combined HR in hormone receptor-positive and ERBB2-postive tumors were 1.02 (95% CI, 0.89–1.15) and 0.95 (95%CI, 0.79–1.14), while the combined HR was 1.26 (95%CI, 1.08–1.48) in TNBC patients (forest plot shown in Figure 6). All the above data were calculated by fixed-effects models. The funnel plot (Figure 7) showed no evidence of publication bias.

## DISCUSSION

Adjuvant chemotherapy (AC) is one of the most important therapies for breast cancer patients. Nevertheless, the optimal time to initiate AC after surgery is still unclear. Due to the potential ethical problems it is unlikely that a prospective clinical trial can be undertaken to explore the association between time delay to initiate AC and survival in breast cancer patients. Moreover, the published randomized controlled clinical trials do not directly suggest the time frame of AC, and the time to initiate AC after surgery ranges from two to 12 weeks [32–35] in different trials. Therefore, the only way to perform our study is to rely on retrospective data. In this meta-analysis the results reveal that OS decreases by 13% and DFS decreases by 14% for every four weeks that AC was delayed. Yu *et al*. [24] reported a similar result: OS decreases by15% and DFS decreases by 16% for every four week delay in the initiation of AC.

Adjuvant chemotherapy decreases the risk of breast cancer mortality mainly through eradication of micrometastatic tumor deposits in breast cancer patients. Some clinical studies suggest that an AC delay to 12 weeks will significantly reduce the effectiveness of systemic therapy. The main theoretical controversies for adverse effects of treatment delay include mathematical models showings that drug resistance mutations can develop over time [36], and from mouse models showing accelerated micrometastatic tumor growth following primary tumor removal [37]. In addition, according to a recent understanding of the significant interplay between the immune system and tumor-produced factors, one could make the assumption that primary tumor removal may reinstate a patient's native antitumor immunity as a result of eliminating the primary source of tumor-mediated immune suppression.

The available studies of the association between survival and the time between surgery to and initiation of AC were included in our meta-analysis. OtheSomer relevant articles were excluded, because these studies were not up to the inclusive criteria. Alkis *et al*. [38], Brooks *et al*. [39] and another Turkish study [20] lacked sufficient data to calculate an adjusted and quantitative HR. Buzdar *et al*. [7], Sanchez *et al*. [9], Shannon *et al*. [10] and Samur *et al*. [40] did not show a worse outcome for patients with AC started later compared to those with AC stared early. A similar meta-analysis by Biagi *et al*. [41] indicated that a 4-week delay initiation of AC might lead to an obvious decrease in OS (HR = 1.06; 95%CI 1.02–1.10) and DFS (HR = 1.08; 95% CI 1.03–1.14) in breast cancer. This study, however, was only an abstract, and it used a fixed-effect model to combine the individual researches studies although a significant heterogeneity between studies did exist.

This meta-analysis shows a detrimental effect on survival when delaying AC. Nevertheless, there was a significant inter-study heterogeneity for OS and DFS. To search for the source of heterogeneity, we performed a subgroup analysis for OS according to the year that the studies were published. After the subgroup analysis, there was no between-study heterogeneity. It showed that the year the study was published was a major source of heterogeneity. Because the studies were published in different years, the clinical methods they used were also different. Moreover, two of the analytical groups (Cold-II [8] and the study by Ke-Da *et al*. [11]) might be the source of heterogeneity. After excluding them, the heterogeneity significantly decreased. We also found that the source of heterogeneity for DFS was the study by Ke-Da. The different results may be caused by the small sample size, patient selection bias, relatively short waiting times, inappropriate WT category classification and use of an unconventional number of cyclesof chemotherapy.

According to breast cancer subtype, Gagliato *et al*. [14] and Chavez-MacGregor *et al*. [17] categorized patients into hormone receptor-positive, ERBB2-positive, and TNBC subgroups. The HRs for OS between WT ≤30 days and 31–60 days in the three subgroups was extracted and analyzed. Results showed that a WT of 31–60 days had no significant impact on patients with ERBB2+ tumors or hormone receptor-positive tumors. While with TNBC, a WT 31–60 days resulted in a 26% increased risk of death. This result could be due to the rapid proliferation rate and aggressive biology of these tumors [42–44]. We did not find a statistically significant adverse effect on OS among patients with ERBB2+ tumors who had a WT 31–60 days. This might be due to the variable use of trastuzumab-based therapy and the small sample sizes in the ERBB2+ category in the two studies (Gagliato *et al*. [14] and Chavez-MacGregor *et al*. [17]).

There are still some limitations to this study. First, all the studies included in this meta-analysis are non random and retrospective. However, it is the only way to perform this type of analysis. Second, not all the prognostic factors we readjusted in our meta-analysis. Other crucial prognostic factors, such as the number of AC cycles, the dose of chemotherapeutic drugs, completion rate for AC, HER2 status and accepting endocrine therapy or not, were not always balanced between the eligible studies. Third, we assume that the effect of WT on survival should be a log-linear relationship. However, the assumption probably didn't conform to the reality. Some studies have shown that if patients initiated AC within 12 weeks after surgery their survival was similar, and those initiating AC at more than 12 weeks had a significant decrease in survival [16, 18]. Therefore, it may be unreliable to use the regressed summary HR across the whole time period to represent the effect of WT on survival. Moreover, the regressed summary HR is unsuitable for extrapolation outside of the time period covered by all the inclusive studies. Fourth, the effect on survival of delaying AC might be different in patients with different clinicopathological features. According to breast cancer subtype, Gagliato *et al*. [14] and Chavez-MacGregor *et al*. [17] performed subgroup analysis on hormone receptor-positive, ERBB2-positive and TNBC tumors. The results showed that a longer time to adjuvant chemotherapy may lead to worse survival in TNBC patients. But we did not find the same conclusion in hormone receptor-positive or ERBB2+ tumors. However, it is insufficient to confirm this result using only two studies, and since there is a lack of individual information, we did not have the opportunity to do comprehensive sub-analyses for all the studies included in this meta-analysis. Fifth, due to the high number of older patients in the studies by Hershman *et al*. [18] and Nurgaliev *et al*. [19], almost 25% of all the patients in our meta-analysis were older than 65 years old. It is unclear whether the age distribution of the patients in this meta-analysis and in the general breast cancer population is the same. If not, it might have a potential impact on the conclusion. Finally, most eligible studies used anthracycline-based and CMF regimens, hence it may be a problem to extrapolate the results of the meta-analysis to the current taxane era.

## CONCLUSIONS

Our results found a significantly unfavorable association between a delay in the initiation of AC and survival of breast cancer patients, especially TNBC patients. The results recommend that the initiation of AC should be optimized by minimizing delay, and WTs for AC should be more strictly controlled for TNBC patients.

## SUPPLEMENTARY MATERIALS FIGURES AND TABLES


