# Supplementary material for: Survival and time to initiation of adjuvant chemotherapy among breast cancer patients: a systematic review and meta-analysis
Source: Oncotarget. 2017 Dec 7;9(2):2739–51. doi: 10.18632/oncotarget.23086 (PMC5788675; doi:10.18632/oncotarget.23086)
Supplement: Supplementary file 1 [file oncotarget-09-2739-s001.pdf]

Survival and time to initiation of adjuvant chemotherapy among breast cancer patients: a systematic review and meta-analysis

SUPPLEMENTARY MATERIALS

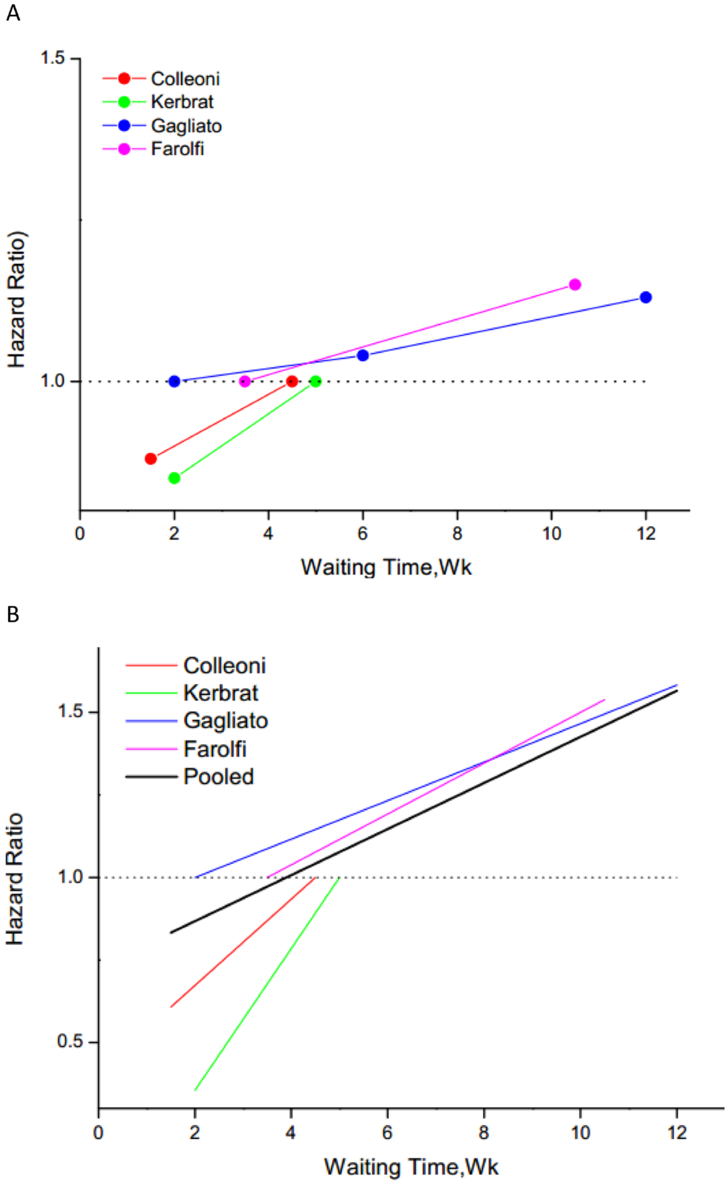

Supplementary Figure 1:Individual hazard ratio for DFS according to waiting time categories. (**A**) The relationship between waiting time categories and overall survival in the 4 independent analytical groups. The hazard ratio (HR) represents a comparison with the lowest waiting time category in each study (as reference). The first author of each study is shown. (**B**) Conversion of HR estimates from the original studies to an HR per week of delay. The slope of each line represents the change in the log HR per week delay. The line for each individual study is located over the range of waiting times.The thick line indicates the weighted average of the HRs from the individual studies. The vertical axis is on a log scale.

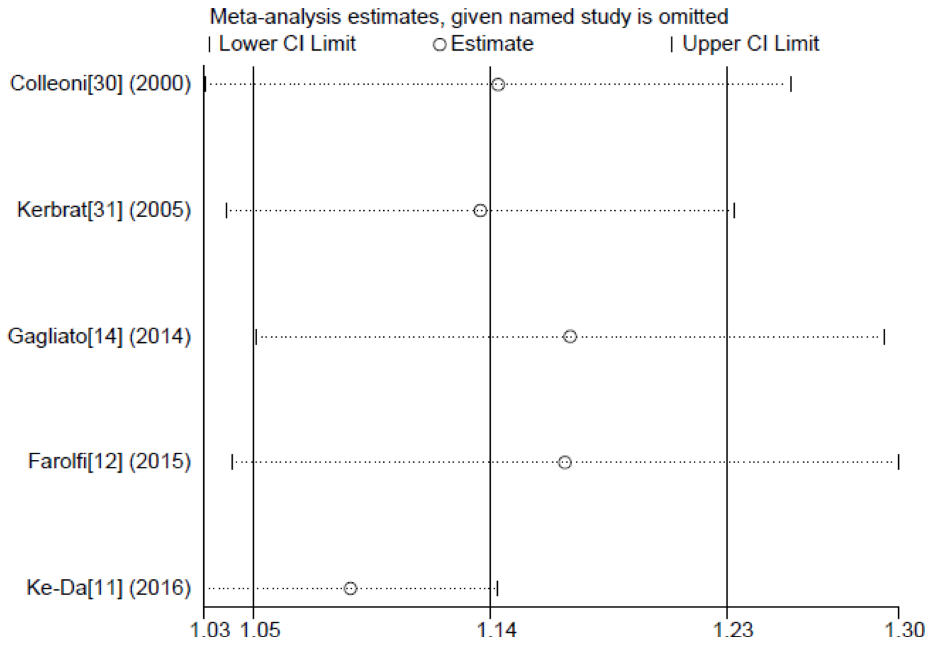

Supplementary Figure 2: Shows the influence of individual studies on the pooled HR for DFS.

Supplementary Table 1: Waiting time to adjuvant chemotherapy and survival in hormone receptor-positive, ERBB2-positive and TNBC subgroups

Source	WT	HR(95%CI)		
	categories	hormone receptor-positive	ERBB2-positive	TNBC
Gagliato *et al*.	≤30 days	Reference	Reference	Reference
	31–60days	1.14 (0.95–1.36)	0.92 (0.69–1.22)	1.74 (1.32–2.29)
	≥61 days	1.29 (1.02–1.64)	1.16 (0.82–0.63))	1.54 (1.09–2.18)
chavez-macgregor *et al.*	≤30 days	Reference	Reference	Reference
	31–60 days	0.90 (0.75–1.08)	0.97 (0.77–1.24)	1.09 (0.90–1.31)
	61–90 days	0.95 (0.77–1.18)	1.03 (0.76–1.38)	1.08 (0.85–1.36)
	≥91 days	1.25 (0.98–1,.59)	1.28 (0.93–1.75)	1.53 (1.17–2.00)
